# Influence of public hesitancy and receptivity on reactive behaviours towards releases of male *Wolbachia*-*Aedes* mosquitoes for dengue control

**DOI:** 10.1371/journal.pntd.0010910

**Published:** 2022-11-11

**Authors:** May O. Lwin, Zoe Ong, Chitra Panchapakesan, Anita Sheldenkar, Li Ting Soh, Irene Chen, Xiaoxi Li, Weixin Niah, Kathryn Vasquez, Shuzhen Sim, Lee-Ching Ng

**Affiliations:** 1 Wee Kim Wee School of Communication and Information, Nanyang Technological University, Singapore; 2 Global Asia, Interdisciplinary Graduate Programme, Nanyang Technological University, Singapore; 3 Affective Computing Group, Institute of High Performance Computing (IHPC), Agency for Science, Technology and Research (A*STAR), Singapore; 4 Environmental Health Institute, National Environment Agency, Singapore; 5 School of Biological Sciences, Nanyang Technological University, Singapore; Faculty of Science, Mahidol University, THAILAND

## Abstract

Singapore, a highly urbanized Asian tropical country that experiences periodic dengue outbreaks, is piloting field releases of male *Wolbachia-*carrying *Aedes aegypti* mosquitoes with the aim of suppressing urban populations of the primary dengue vector *Aedes aegypti*. This study proposes and assesses a model to explain the roles of hesitancy and receptivity towards Project *Wolbachia*–Singapore in influencing reactive mosquito prevention behaviors (reactive behaviors) towards the release of *Wolbachia-Aedes* mosquitoes for residents living in the release sites. Interestingly, both hesitancy and receptivity predicted greater instances of reactive behaviors. The model also examines the roles of general knowledge about *Wolbachia* technology, perceived severity of mosquito bites, perceived density of mosquitoes, and social responsibility as predictors of hesitancy, receptivity, and reactive behaviors towards the release of *Wolbachia-Aedes* mosquitoes. Hesitancy towards the project mediated the effects of general knowledge, perceived severity of mosquito bites, and perceived density of mosquitoes on reactive behaviors towards the releases, although receptivity towards the project did not. Having less knowledge about Project *Wolbachia*–Singapore was associated with higher hesitancy towards the project and higher likelihood of performing reactive behaviors towards the releases. Individuals who perceive mosquito bites to be more severe and think that there are more mosquitoes in their living environments were also more likely to be hesitant about the project and practice reactive behaviors. However, both hesitancy and receptivity towards the project mediated the effect of social responsibility on reactive behaviors. Receptivity towards the project was driven by social responsibility, which was also associated with reduced hesitancy towards the project. Our findings suggest that, to address the hesitancy reported by a minority of participants, future outreach efforts should focus on strengthening the public’s sense of social responsibility and on tailored education campaigns targeting groups with low levels of knowledge of the project.

## Author summary

The incidence of dengue, a serious mosquito-borne disease, has escalated globally in recent decades. Prevention is critical for reducing the incidence of dengue, and one way of doing so is by controlling the vector population. Singapore has been piloting the use of field releases of male *Wolbachia*-carrying *Aedes* mosquitoes to suppress the vector *Aedes* mosquito population (Project *Wolbachia*–Singapore), but the public’s perception and social impact of this community-based method is not well understood. To address this gap, this study sought to identify and explain the drivers and outcomes of the public’s perceptions towards Project *Wolbachia*–Singapore. Findings reveal that different combinations of individual characteristics predict for receptivity towards Project *Wolbachia*–Singapore, hesitancy towards Project *Wolbachia*–Singapore, and the tendency to behave reactively to the release of *Wolbachia*-carrying *Aedes* mosquitoes. These findings also suggest ways of increasing receptivity and reducing hesitancy towards such community-based prevention efforts, which can in turn help to ensure the success of such projects.

## Introduction

The incidence of dengue, a mosquito-borne disease transmitted by *Aedes* mosquitoes, has escalated globally in recent decades [1]. Singapore is hyperendemic for dengue and experiences periodic dengue outbreaks [2], with an estimated average economic burden of at least US$1.014 billion and disease burden of at least 7,645 DALYs from 2010 to 2020 [3]. Dengue is thus a key area of concern in the local context. While a dengue vaccine is commercially available, its use is limited due to safety concerns [1,4,5]. As such, much of the fight against dengue continues to rest on stakeholders’ efforts and vector control [1,6].

### Dengue control and Project *Wolbachia–*Singapore

In Singapore, dengue prevention is spearheaded by the National Environment Agency (NEA), which runs an integrated dengue control program comprising surveillance, preventive source reduction, outbreak management, and community engagement. As a result of vector control and public education efforts [6], *Aedes* breeding sites are now only detected in about 2% of properties inspected in Singapore [7]. Despite this low *Aedes* House Index, Singapore continues to experience dengue outbreaks. This is likely due to a combination of factors, including reduced herd immunity following decades of effective vector control [8], changes in the predominant circulating dengue virus serotype [2], and variations in environmental conditions such as temperature and rainfall [9]. As part of efforts to develop and evaluate novel dengue control tools, since 2016, Singapore has been piloting field releases of male *Wolbachia*-carrying *Aedes aegypti (Wolbachia-Aedes)* mosquitoes to suppress urban *Aedes aegypti* mosquito populations—an initiative termed Project *Wolbachia*–Singapore [10,11].

In line with WHO’s advice for carefully planned pilot deployment of *Wolbachia*-related technology, NEA has launched Project *Wolbachia*–Singapore in phases, accompanied by surveillance and reporting of the findings at each stage [10,12]. Thus far, the project has achieved up to 98% suppression of the *Aedes aegypti* mosquito population [10] and up to 88% reduction of dengue incidences at the two core study sites with at least one year of releases in Yishun and Tampines [11]. The success of the *Wolbachia* suppression technology in reducing vector populations has also been demonstrated in pilots in other countries, such as USA, China, and Thailand [13–15]. However, less is understood about the social impact of *Wolbachia* technology on communities.

### Public perceptions of *Wolbachia* technology

The handful of studies examining public perceptions of dengue-related applications of *Wolbachia* have predominantly focused on programs which aim to control dengue through stable establishment of *Wolbachia* in the field mosquito population by releasing both male and female mosquitoes [16–18]. For people living in potential *Wolbachia-Aedes* release sites in Australia, safety of the technology, presence of regulatory oversight, and efforts to engage the community were crucial for acceptance of the technology [16]. In comparison, a study by Azil et al. [17] conducted in Malaysia prior to the release of *Wolbachia-Aedes* mosquitoes found good acceptance of *Wolbachia* amongst healthcare staff, and this was associated with having a better science background and having good knowledge about dengue. Arham et al. [18] also found a positive attitude toward the use of *Wolbachia-Aedes* mosquitoes amongst the general population in Klang Valley, Malaysia, which was associated with factors such as trust in the authorities, perceived benefits of *Wolbachia-Aedes* mosquitoes, and religiosity.

Thus far, only very few studies have provided insights into the public’s perception about the release of only male *Wolbachia-Aedes* mosquitoes to suppress vector populations [13,19,20]. Kittayapong et al. [13] observed that almost all participants were willing to accept the release of male *Wolbachia*-*Aedes* mosquitoes in or near their homes. Those who objected to the release of male *Wolbachia*-*Aedes* mosquitoes in their homes were older, less educated, lacked an understanding of the difference in biting ability between male and female mosquitoes, and were afraid of being bitten by mosquitoes. Similarly, in Singapore, surveys conducted prior to and during the initial phase of Project *Wolbachia* found high levels of support for male *Wolbachia-Aedes* releases, and household perception surveys conducted during the initial phase of Project *Wolbachia* revealed that the majority of households in the study sites did not have objections to the release of male *Wolbachia—Aedes* mosquitoes in their estates [19]. A more recent publication [20] examining public awareness, knowledge, and perception of Project *Wolbachia–*Singapore, found that demographic factors like age, education levels, and length of exposure to Project *Wolbachia*–Singapore can affect awareness, knowledge and acceptance of the project.

The dearth of studies on public perceptions and the community impact of *Wolbachia*-based dengue preventive measures, particularly the suppression strategy, is a critical gap in the literature. Since the Project *Wolbachia–*Singapore pilot involves the release of male *Wolbachia*-*Aedes* mosquitoes in close proximity to residential dwellings, understanding public perceptions and the social impact of the project is necessary for its long-term success. Moreover, understanding the drivers of public perceptions and behaviors can help to identify ways of increasing the project’s success. As such, we aim to examine how individual characteristics (beyond demographic factors) shape public attitudes and behaviors in response to Project *Wolbachia–*Singapore.

### Theoretical framework and research questions

To examine the public perceptions and community impact of Project *Wolbachia–*Singapore, this study built a model based mainly on two theoretical frameworks frequently used in studies of dengue–the knowledge, attitude, and practice (KAP) model [21–26], and the Health Belief Model (HBM) [27–30]. Expanding the KAP framework with other variables such as those from the HBM provides a more comprehensive perspective on potential predictors of the public’s reactive mosquito prevention behaviors in the context of *Wolbachia* suppression strategy.

The KAP model posits that more knowledge leads to more positive attitudes, which in turn leads to better practices or behaviors. Indeed, various studies have found significant positive correlations between knowledge and attitudes, knowledge and preventive behaviors, as well as attitudes and preventive behaviors in the context of dengue [21–23]. However, previous studies have also proposed that dengue prevention practices are not influenced only by knowledge and attitude and suggested the need to examine other potential contributing factors, such as those involved in motivation [24–26].

The HBM is a theoretical framework that seeks to explain the motivations behind health behaviors. It theorizes that individuals will only take preventive action against a disease if they believe in their susceptibility to the disease, the severity of the disease, and the effectiveness of the preventive measures [30]. Indeed, studies that have examined predictors of preventive behaviors towards dengue using HBM have found that participants who perceived higher threat (i.e. severity, susceptibility) and density of mosquitoes in their neighborhood were more likely to undertake dengue prevention practices than their counterparts [28,29]. As such, this study also included the perceived severity of mosquito bites and perceived density of mosquitoes as additional independent variables.

Beyond these KAP and HBM variables, other factors that have also been examined as predictors of attitudes towards dengue prevention include demographic variables (age and education level), experience with dengue, and religiosity [18,20–22]. The role of social responsibility, however, has yet to be examined. Although the production and release of male *Wolbachia*-*Aedes* mosquitoes is led and managed by a government body, the initiative’s sustainability is highly dependent on public support and tolerance for the release of the mosquitoes. Having a strong sense of social responsibility could drive one to bear the potential inconvenience associated with the release of mosquitoes for the greater community benefit. Thus, social responsibility was also included as an independent variable in our study.

An earlier study on Project *Wolbachia*–Singapore [20] found that the public generally had positive attitudes regarding Project *Wolbachia*–Singapore. The majority of the participants trusted NEA with the project and were willing to accept more mosquitoes in their living areas due to Project *Wolbachia*–Singapore for at least half a year. However, some studies have suggested that positive and negative attitudes are functionally separate entities that can have different contributive factors and effects on behavior [31,32]. Therefore, to delve deeper into public attitudes towards Project *Wolbachia*–Singapore, attitudes were grouped into two sub-constructs in this study–hesitancy towards Project *Wolbachia*-Singapore and receptivity towards Project *Wolbachia*–Singapore.

**Fig 1:**
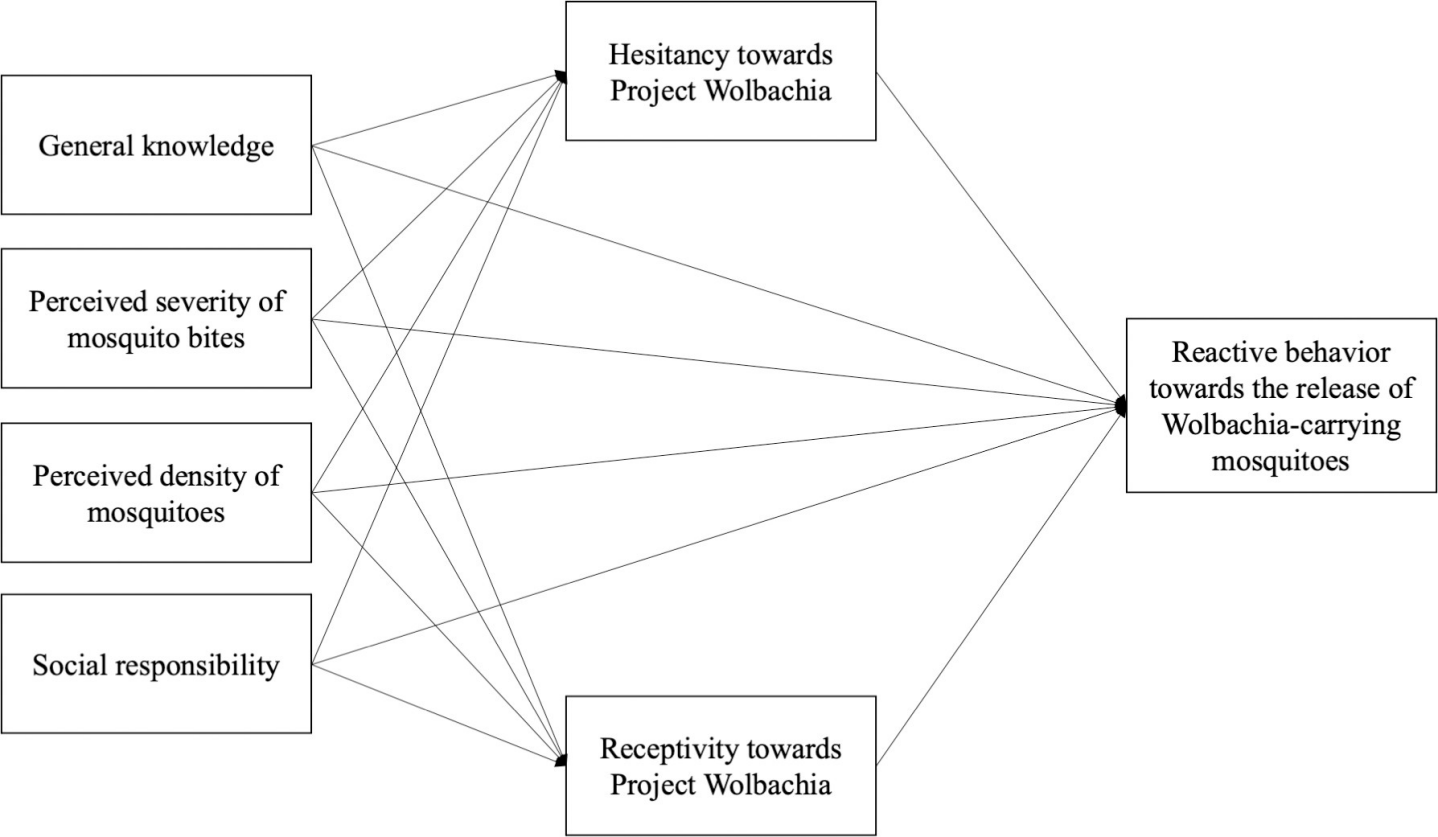
Proposed conceptual model: Public *Wolbachia* Response Framework.

Past findings on the relationships between this study’s variables have been inconsistent in previous studies examining dengue-related knowledge, attitudes and behaviors. For example, while some studies reported significant positive correlations between knowledge and attitudes, knowledge and preventive behaviors, as well as attitudes and preventive behaviors [21–23]; others found at least one of these three relationships to be non-significant [17,33]. The effects of newly added variables such as severity of mosquito bites, perceived density of mosquitoes and social responsibility are also unclear. Therefore, further investigation is needed to understand the relationships between these variables more clearly, to explain the roles of hesitancy and receptivity towards Project *Wolbachia*–Singapore in influencing reactive mosquito prevention behaviors towards the release of *Wolbachia-Aedes* mosquitoes for residents living in the release sites. To examine these in detail, we propose a conceptual model where general knowledge, perceived severity of mosquito bites, perceived density of mosquitos and social responsibility all influence reactive behaviors towards the release of *Wolbachia-Aedes* mosquitoes, and some of their effects are mediated through hesitancy and receptivity towards Project *Wolbachia*–Singapore (Fig 1).

Our conceptual model (Fig 1: Public *Wolbachia* Response Framework) proposes that reactive mosquito prevention behaviors towards the release of *Wolbachia-Aedes* mosquitoes can be driven by both primary and secondary predictors. We propose that hesitancy (unwillingness to accept the release of *Wolbachia-Aedes* mosquitoes) and receptivity (willingness to accept the release of *Wolbachia-Aedes* mosquitoes) towards Project *Wolbachia*–Singapore are the primary drivers of reactive behaviors. In comparison, general knowledge about Project *Wolbachia*–Singapore and *Wolbachia* technology, perceived severity of mosquito bites, perceived density of mosquito bites, and social responsibility are secondary predictors. These secondary predictors can either affect the likelihood of reactive behaviors directly, or indirectly through their effect on the primary predictors of hesitancy and receptivity. In other words, we expect that hesitancy and receptivity mediate or act as central gatekeeping variables (mediators) between the secondary predictors and reactive mosquito prevention behaviors.

Drawing upon these proposed relationships, this study aims to address four research questions. First, we aim to examine the direct effect of secondary predictors on primary predictors and reactive mosquito prevention behaviors (reactive behaviors):

RQ1: How will participants’ general knowledge about Project *Wolbachia*–Singapore and *Wolbachia* technology, perceived severity of mosquito bites, perceived density of mosquitoes and social responsibility drive (a) hesitancy towards Project *Wolbachia*–Singapore, (b) receptivity towards Project *Wolbachia*–Singapore and (c) reactive behaviors towards the release of male *Wolbachia-Aedes* mosquitoes?

Second, we aim to examine the direct effect of primary predictors on reactive mosquito prevention behaviors (reactive behaviors):

RQ2: How will participants’ (a) hesitancy towards Project *Wolbachia*–Singapore and (b) receptivity towards Project *Wolbachia*–Singapore drive reactive behaviors towards the release of male *Wolbachia-Aedes* mosquitoes?

Third, we aim to examine the indirect effect of secondary predictors on reactive mosquito prevention behaviors (reactive behaviors) through the primary predictor of hesitancy:

RQ3: How does hesitancy towards Project *Wolbachia*–Singapore mediate the effect of secondary predictors ((a) general knowledge about Project *Wolbachia*–Singapore, (b) perceived severity of mosquito bites, (c) perceived density of mosquitoes and (d) social responsibility) on reactive behaviors towards the release of male *Wolbachia-Aedes* mosquitoes?

Fourth, we aim to examine the indirect effect of secondary predictors on reactive mosquito prevention behaviors (reactive behaviors) through the primary predictor of receptivity:

RQ4: How does receptivity towards Project *Wolbachia*–Singapore mediate the effect of secondary predictors ((a) general knowledge about Project *Wolbachia*–Singapore, (b) perceived severity of mosquito bites, (c) perceived density of mosquitoes and (d) social responsibility) on reactive behaviors towards the release of male *Wolbachia-Aedes* mosquitoes?

## Methods

### Ethics statement

The study protocol was reviewed by the National Environment Agency Bioethics Review Committee (BRC) which deemed the project to be surveillance in nature and therefore exempted from formal bioethics review. The project was designed to guide public health policies, programme and actions to prevent and diseases. The survey was only administered if informed verbal consent was obtained.

### Survey

A survey was conducted with 500 residents in the two Singapore neighborhoods that served as pilot sites for Project *Wolbachia*. Surveys were administered in person using a door-to-door approach via FormSG [34], an online form building tool developed for use within Singapore Government institutions, through an iPad. More details about the data collection can be found in an earlier publication [20].

### Measures

Details about the items used for each measure are presented in Table 1.

**Table 1:** Measure used for each factor.

Measure	Items
General knowledge about Project *Wolbachia–*Singapore and *Wolbachia* technology*	• *Wolbachia* is a bacterium [True] • *Wolbachia* is safe [True] • All mosquitoes regardless to their gender could bite [False] • *Wolbachia-Aedes* suppression targets many species of mosquitoes [False] • *Wolbachia-Aedes* mosquito is not genetically modified [True] • Not all mosquitoes transmit dengue equally [True] • Mating between *Wolbachia-Aedes* males and wildtype urban females result in eggs that do not hatch [True] • Project *Wolbachia*–Singapore is being deployed all over Singapore [False] • Project *Wolbachia*–Singapore involves the release of both male and female *Wolbachia*-carrying *Aedes* mosquitoes. [False] • Male *Wolbachia-*carrying *Aedes* mosquitoes can help reduce dengue mosquito population. [True] • We need to release male *Wolbachia-Aedes* only once to effectively reduce dengue mosquito population in the long term. [False]
Perceived severity of mosquito bites	• Getting mosquito bites will increase my risk of getting serious diseases • Getting mosquito bites will affect my ability to do my usual activities
Perceived density of mosquitoes	• I’ve seen more mosquitoes in my house recently (past 1 month) • There are more mosquitoes in my neighborhood recently • I’ve gotten more mosquito bites in my house recently
Social responsibility	• I believe that all residents have a responsibility to support the Project *Wolbachia*-Singapore by NEA though it may temporarily cause inconvenience to their daily lives • I believe that I have the responsibility as a resident to support the Project *Wolbachia*-Singapore by NEA
Hesitancy towards Project *Wolbachia–Singapore*	• The release of *Wolbachi*a-carrying mosquitoes without consent of residents is unethical. • Project *Wolbachia*–Singapore is a waste of money • The Project *Wolbachia*–Singapore is unnecessary • The release of *Wolbachia*-carrying mosquitoes is disturbing my daily life • There is no need to release *Wolbachia*-*Aedes* if there is no mosquito-borne infectious disease outbreak • NEA should release the male *Wolbachia*- *Aedes* only if the community demands it • NEA should release the *Wolbachia-Aedes* only in areas that have disease outbreaks
Receptivity towards Project *Wolbachia*–Singapore	• NEA should release the *Wolbachia-Aedes* in more areas to prevent mosquito-borne infectious diseases • NEA should release the *Wolbachia-Aedes* in the whole of Singapore. • NEA should promote the Project *Wolbachia*–Singapore more than they have been doing
Reactive behavior towards the release of *Wolbachia*- *Aedes* mosquitoes	• Concerns about the release of *Wolbachia-Aedes* are encouraging me to (a) use mosquito nets (b) insect repellent/mosquito repelling plants (c) close windows and doors (d) carry out weekly checks for stagnant water • I hunt and kill every mosquito I see in my house

### General knowledge about Project *Wolbachia*–Singapore and *Wolbachia* technology

General knowledge about Project *Wolbachia*–Singapore and *Wolbachia* technology were measured using 11 items (e.g. *Wolbachia* is a bacterium [True]), which were expanded from the 4 items used in an online public sentiment survey conducted prior to the implementation of Project *Wolbachia*–Singapore [19]. These 11 items were also used as a composite measure of General Knowledge scores in [20].

### Perceived severity of mosquito bites

Perceived severity of getting bitten by mosquitoes was measured using two items (e.g. Getting mosquito bites will increase my risk of getting serious diseases) adapted from Kwong and Lam’s 2008 study on health beliefs about influenza vaccination [35] on a 5-point Likert scale (1 = Strongly disagree, 5 = Strongly agree). These two items were significantly correlated (*r* = .510, *p* < .01).

### Perceived density of mosquitoes

Perceived geographical density of mosquitoes was measured using three items (e.g. There are more mosquitoes in my neighborhood recently) adapted from the questions used in a mobile application designed to monitor perceptions of mosquito abundance and nuisance [36] on a 5-point Likert scale (1 = Strongly disagree, 5 = Strongly agree). Internal consistency of these three items was also good (α = .814).

### Social responsibility

Social responsibility was measured using two items (e.g. I believe that I have the responsibility to support the Project *Wolbachia*–Singapore by NEA) adapted from Doolittle and Faul’s civic engagement scale [37], on a 5-point Likert scale (1 = Strongly disagree, 5 = Strongly agree). The two items were significantly correlated (*r* = .701, *p* < .01).

### Hesitancy towards Project *Wolbachia*–Singapore

Hesitancy towards Project *Wolbachia*–Singapore was measured using self-created seven items (e.g. Project *Wolbachia*–Singapore is unnecessary) on a 5-point Likert scale (1 = Strongly disagree, 5 = Strongly agree). Internal consistency of these seven items was also good (α = .746).

### Receptivity towards Project *Wolbachia*-Singapore

Receptivity towards Project *Wolbachia*–Singapore towards the implementation of Project *Wolbachia*–Singapore was measured using self-created three items (e.g. NEA should release the *Wolbachia-Aedes* in the whole of Singapore) on a 5-point Likert scale (1 = Strongly disagree, 5 = Strongly agree). Internal consistency of these three items was also acceptable (α = .665).

### Reactive behavior towards the release of *Wolbachia-Aedes aegypti* mosquitoes

The likelihood of carrying out reactive behavior towards the release of *Wolbachia-Aedes* mosquitoes was measured using five items (e.g. Concerns about the release of *Wolbachia-Aedes* are encouraging me to use mosquito nets) adapted from measures of dengue preventive practices [21,23,28] on a 5-point Likert scale (1 = Strongly disagree, 5 = Strongly agree). In this study reactive behavior refers to preventive measures taken against mosquitoes due to the release of *Wolbachia-Aedes* mosquitoes. Internal consistency of these five items was also acceptable (α = .681).

### Data analysis

IMB SPSS Statistics version 27 was used to assess the reliability of the measures (Armonk, NY, USA). For path analysis, we used IMB SPSS Amos version 27 (Armonk, NY, USA). The specific indirect effects of general knowledge, perceived severity of mosquito bites, perceived density of mosquitoes, and social responsibility on reactive behaviors through hesitancy and receptivity were calculated by inputting each relationship of interest as new user-defined estimands (e.g. the effect of general knowledge on hesitancy multiplied by the effect of hesitancy on reactive behaviors). There were no missing data amongst the participants for the variables included in this study. Bootstrapping was performed for 5000 bootstrap samples at 95% bias-corrected confidence level. Data is available on request from the authors.

## Results

A total of 500 participants residing in two study sites within Singapore were surveyed. Further socio-demographic details of the participants can be found in [20].

### Public attitudes towards Project *Wolbachia–*Singapore

The study results showed that in general, participants have a positive outlook towards Project *Wolbachia*–Singapore, leaning towards being more receptive (M = 3.61, SD = 0.660) towards the project. Specifically, 72.6% of participants are receptive towards Project *Wolbachia*–Singapore, 18.4% are neutral, and a small minority (9%) of participants are not receptive of the project. When participants were directly asked about their hesitancy towards the project, only 18.2% of participants reported being hesitant.

### Path analysis

**Fig 2:**
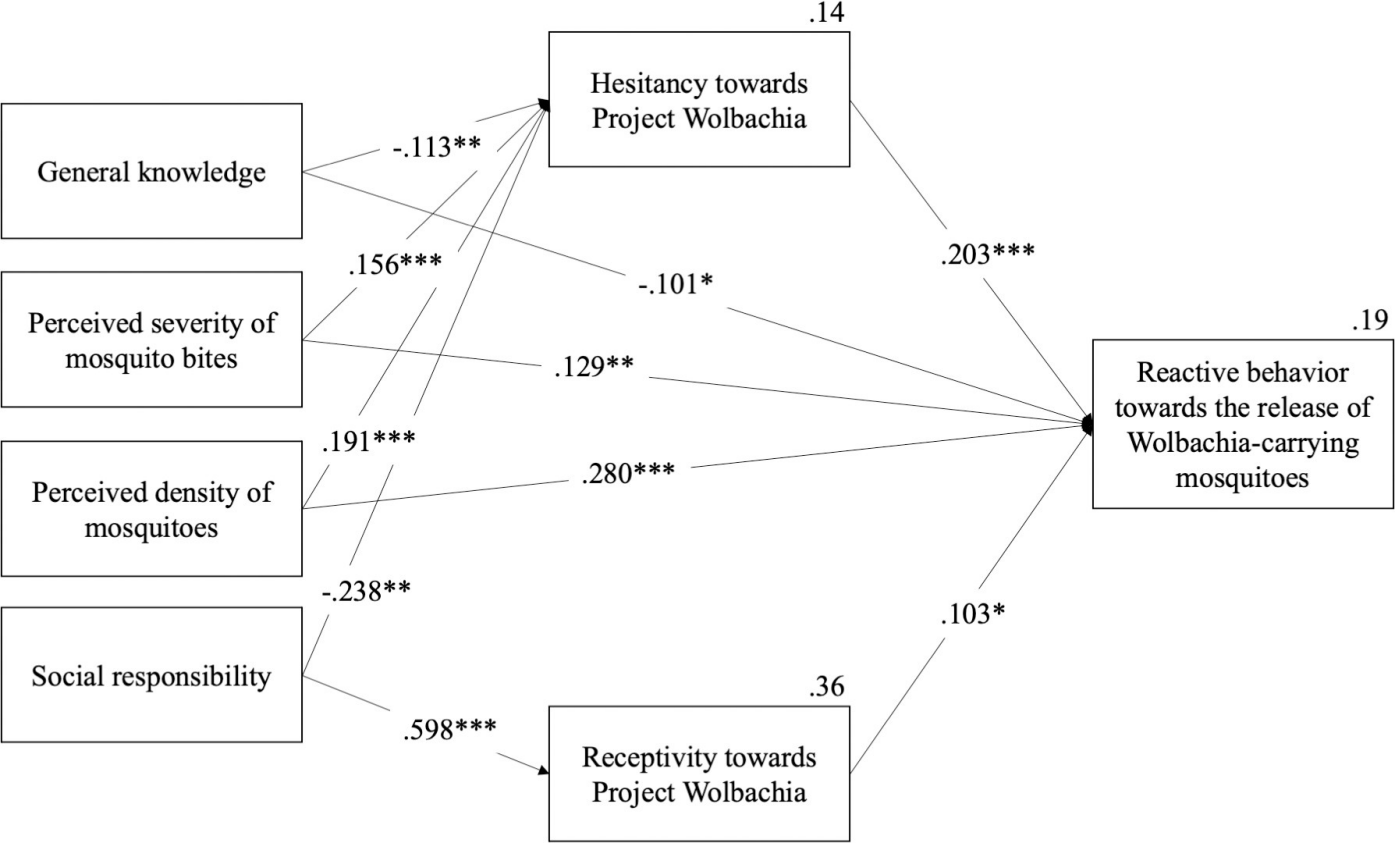
Public *Wolbachia* Response Framework: Predictors of reactive behavior towards release of *Wolbachia*-carrying mosquitoes. Significant pathways are shown with their standardized regression coefficients for direct effects. *p < .05, **p < .01, ***p < .00.

Path analysis revealed that the initial conceptual model was overfitting. Removal of non-significant paths resulted in a good model fit χ2(5) = 13.914, relative χ2 (CMIN/df) = 2.783, *p* = 0.016, CFI = .980, TLI = .916, RMSEA = .060, PCLOSE = .283, SRMR = .0228, GFI = .992, AGFI = .956, NFI = .970. Although χ^2^ was significant, this could be due to large sample size (n>200) (Schumacker & Lomax, 2016). The final model is shown in Fig 2.

### Direct effect of secondary predictors on primary predictors and reactive behaviors

Higher general knowledge about Project *Wolbachia–*Singapore and *Wolbachia* technology predicted less hesitancy about the project (*β* = -.113, *p* < .01) and lower likelihood of performing reactive behaviors due to concerns regarding the release of male *Wolbachia*-*Aedes* mosquitoes (*β* = -.101, *p* < 0.05). In contrast, both higher perceived severity of mosquito bites and higher perceived density of mosquitoes in one’s living environment predicted greater hesitancy towards Project *Wolbachia–*Singapore (perceived severity of mosquito bites: *β* = .156, *p* < .001; perceived density of mosquitoes: *β* = .191, *p* < .001) and higher likelihood of performing reactive behaviors in response to concerns regarding the release of male *Wolbachia*-*Aedes* mosquitoes (perceived severity of mosquito bites: *β* = .129, *p* < .01; perceived density of mosquitoes: *β* = .280, *p* < .001). These three predictors, however, did not have a significant relationship with receptivity towards Project *Wolbachia–*Singapore. The last predictor, sense of social responsibility, showed a different pattern of effect on the outcome variables. Having a stronger sense of social responsibility predicted both less hesitancy (*β* = -.238, *p* < .01) and greater receptivity towards Project *Wolbachia*–Singapore (*β* = .598, *p* < .001). But an individual’s sense of social responsibility did not have a significant relationship with their likelihood of carrying out reactive behaviors out of concern about male *Wolbachia-Aedes* mosquitoes, answering our RQ1.

### Direct effect of primary predictors on reactive behaviors

Both hesitancy (*β* = .203, *p* < .001) and receptivity (*β* = .103, *p* < .05) towards Project *Wolbachia–*Singapore predicted for a greater likelihood of performing reactive behaviors due to the presence of more mosquitoes, answering our RQ2.

### Indirect effect of secondary predictors on reactive behaviors through primary predictors

**Fig 3:**
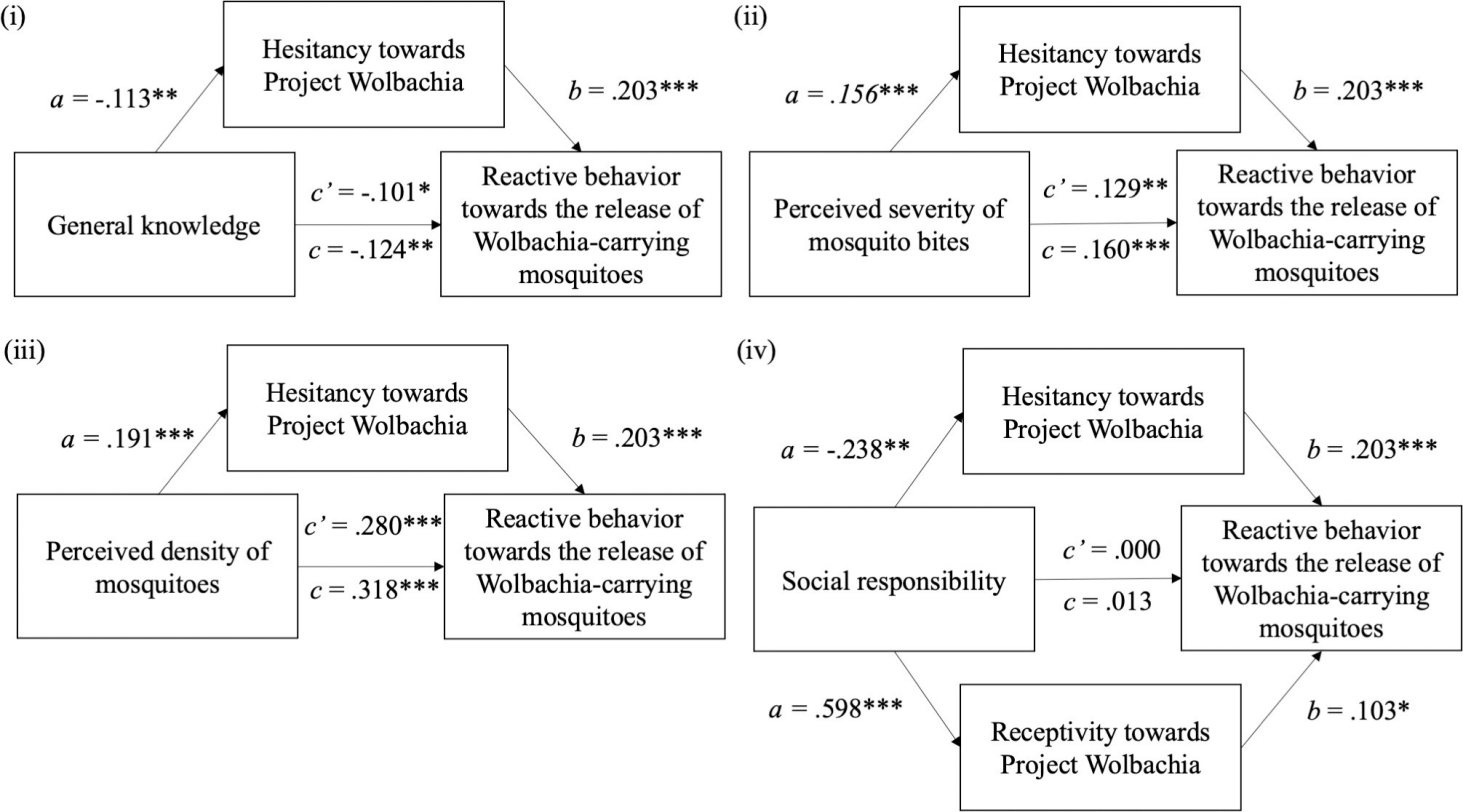
Roles of hesitancy towards Project *Wolbachia*–Singapore and receptivity towards Project *Wolbachia*–Singapore as mediators. Each numerical value is the standardized regression coefficient for that pathway. *a* refers to the direct effect of predictor on the mediator, *b* refers to the direct effect of the mediator on the outcome, *c’* refers to the direct effect of the predictor on the outcome, while *c* refers to the total effect (direct + indirect effect of the predictor on the outcome).

Fig 3i shows that hesitancy about Project *Wolbachia–*Singapore mediated the effect of knowing more about the project on the likelihood of performing reactive behaviors due to concerns about the release of male *Wolbachia-Aedes* mosquitoes. Specifically, higher general knowledge about Project *Wolbachia–*Singapore predicted less hesitancy towards the project, which in turn predicted lower likelihood of behaving reactively towards male *Wolbachia-Aedes* mosquitoes (Standardized indirect effect = -.023, SE = 0.010, *p* < .01, 95% CI [-0.047, -0.006]). Hesitancy towards Project *Wolbachia–*Singapore also mediated the effects of one’s perception about the severity of mosquito bites (Fig 3ii) and the presence of mosquitoes in one’s living environment (Fig 3iii) on the likelihood of performing reactive behaviors due to concerns about the release of male *Wolbachia-Aedes* mosquitoes. Perceiving mosquito bites to have serious consequences predicted greater hesitancy towards Project *Wolbachia–*Singapore, and this in turn predicted a greater likelihood of behaving reactively to concerns about male *Wolbachia-Aedes* mosquitoes (Standardized indirect effect = .032, SE = 0.011, *p* < .001, 95% CI [0.014; 0.057]). Similarly, feeling like there were more mosquitoes in one’s living environment predicted greater hesitancy about the project and in turn a greater likelihood of performing reactive behaviors against mosquitoes (Standardized indirect effect = .039, SE = 0.012, *p* < .001, 95% CI [0.019; 0.067]). In contrast, the total indirect effect of social responsibility on reactive behaviors due to concerns about male *Wolbachia-Aedes* mosquitoes through the two mediators, hesitancy and receptivity towards Project *Wolbachia*–Singapore, was not significant (Fig 3iv). However, further analysis of the specific mediating effect of hesitancy and receptivity respectively revealed that each of these indirect paths was significant on its own. That is, having a stronger sense of social responsibility predicted less hesitancy towards Project *Wolbachia*–Singapore and in turn a lower likelihood of behaving reactively due to concerns about *Wolbachia-Aedes* mosquitoes (Specific indirect effect = -.051, SE = 0.15, *p* < .001, 95% CI [-.083, -.025]). Having a stronger sense of social responsibility also predicted greater receptivity towards Project *Wolbachia*–Singapore and in turn a greater likelihood of behaving reactively due to concerns about *Wolbachi*a-*Aedes* mosquitoes (Specific indirect effect = .065, SE = .027, *p* < .05, 95% CI [.013, .119]). On the whole, the data indicates that while receptivity and hesitancy towards Project *Wolbachia*–Singapore partially mediated the effect of general knowledge, perceived severity of mosquito bites and perceived density of the mosquitoes on the likelihood of performing reactive behaviors, the effect of social responsibility on the likelihood of performing reactive behaviors is completely mediated by receptivity and hesitancy towards Project *Wolbachia*–Singapore, answering RQ3 and RQ4.

## Discussion

This research set out to develop a model that explains the specific roles of hesitancy towards Project *Wolbachia*–Singapore versus receptivity towards Project *Wolbachia*–Singapore in influencing the public’s reactive behaviors towards release of male *Wolbachia-Aedes* mosquitoes in Singapore—a less explored area of research in the vector control field given the novelty of the *Wolbachia* technology. This study contributes several important insights with both theoretical and practical implications. Overall, our findings revealed that most residents in the *Wolbachia* release sites were receptive towards the project, which is in line with earlier studies of Project *Wolbachia*–Singapore [19,20]. Together with the efficacy of the technology at reducing mosquito populations and dengue cases [11], residents’ receptivity towards Project *Wolbachia*–Singapore supports the feasibility of further expanding this project.

In examining the factors that drive the public’s attitudes and behaviors in relation to Project *Wolbachia–*Singapore, receptivity towards Project *Wolbachia*–Singapore, hesitancy towards Project *Wolbachia*–Singapore, and the likelihood of performing reactive behaviors due to concerns about the release of male *Wolbachia-Aedes* mosquitoes were found to be driven by different combinations of the four secondary predictors examined (general knowledge, perceived severity of mosquito bites, perceived density of mosquitoes and social responsibility). Both primary predictors, receptivity towards Project *Wolbachia*–Singapore and hesitancy towards Project *Wolbachia*–Singapore, were also found to predict for the likelihood of performing reactive behaviors. The positive association between hesitancy towards Project–*Wolbachia* Singapore and the likelihood of performing reactive behaviors is unsurprising, as the minority who are more hesitant may be less trustful of the initiative’s ability to tackle dengue and therefore take it upon themselves to be more vigilant against mosquitoes. In contrast, the positive association between receptivity and the likelihood of performing reactive behaviors may seem counterintuitive. However, being receptive towards Project *Wolbachia*–Singapore and taking precautions against mosquitoes are not mutually exclusive. For example, while an individual may welcome Project *Wolbachia*–Singapore as a way to tackle dengue, they can also continue to be vigilant about protecting themselves from mosquitoes by performing the necessary preventive behaviors. NEA has indeed taken care to convey to the residents in the Project *Wolbachia*–Singapore release areas that they should continue to do so as there may be other species of mosquitoes such as *Aedes albopictus* and *Culex spp*. Which can contribute to mosquito biting and are not affected by the project.

On the theoretical front, this study extended the KAP model by demonstrating that other factors like perceived severity of mosquito bites, perceived density of mosquitoes, and social responsibility can also contribute to attitudes and behaviors in this context of using *Wolbachia* technology to prevent dengue. The study also reveals that there is merit in conducting a deeper analysis of attitudes. As suggested by previous studies [31,32], positive attitudes and negative feelings appear to be functionally separate entities in our study context of Project *Wolbachia–*Singapore. While both hesitancy and receptivity towards the project are significant predictors of reactive behavior towards the release of male W*olbachia*-*Aedes* mosquitoes, the contributing factors for these two constructs are different. These differences might have been masked if we had used an overall measure of attitude. To the best of our knowledge, only one study on *Wolbachia* technology in Malaysia focused on a particular (positive) aspect of attitude as “acceptance of dengue biological control” [17]. It might therefore be interesting to reexamine factors that have been discovered to influence overall attitude, such as demographic factors, experience with dengue, trust, and religiosity [18,22,23,38] to determine their effect on positive attitudes and negative feelings as separate constructs.

The different combinations of factors that predict for receptivity towards Project *Wolbachia*–Singapore, hesitancy towards Project *Wolbachia*–Singapore and tendency to behave reactively towards the release of *Wolbachia-Aedes* mosquitoes also have practical implications. Of the four secondary predictors examined, only social responsibility was significantly associated with both receptivity (positive association) and hesitancy (negative association) towards Project *Wolbachia*–Singapore. Those who are more socially responsible are likely to be more supportive of initiatives meant to benefit the community at large, even if it might bring about some inconvenience for them, while those who are less socially responsible are more likely to be hesitant about supporting these initiatives. As the only factor that simultaneously increases receptivity while decreasing hesitancy towards Project *Wolbachia*–Singapore, focusing on the idea of social responsibility in future campaigns would likely be useful in helping to improve public acceptance of Project *Wolbachia*–Singapore as it continues to expand.

Although the generally positive public perception about Project *Wolbachia*–Singapore is encouraging, the minority of respondents who are more hesitant about Project *Wolbachia*–Singapore should not be overlooked. Our study highlights various ways through which hesitancy towards such projects can potentially be reduced. Other than appealing to the public’s sense of social responsibility, increasing general knowledge about Project *Wolbachia*–Singapore is also an important way of reducing hesitancy towards the project. While a prior study by Azil et al. [17] reported a positive association between knowledge about dengue and acceptance of the stable establishment of *Wolbachia*, this study contributes new information by examining the influence of knowledge about Project *Wolbachia*–Singapore (including the *Wolbachia* technology) on attitudes towards the use of *Wolbachia* technology for suppression of the mosquito vector population. The negative association identified between knowledge and hesitance towards Project *Wolbachia*–Singapore in this study indicates the importance of improving knowledge about Project *Wolbachia*–Singapore. Ensuring good public knowledge of the technology has been an integral part of the project since its inception, with communication and outreach campaigns conducted since 2016 with growing intensity as the project expands. To build on these existing efforts, it might be useful to characterize those who are less knowledgeable about the project so that future education outreach efforts may be tailored for them.

Another key finding is that people who think there are more mosquitoes in their living environments and people who perceive mosquito bites to be more severe were more likely to practice reactive behaviors, and this was in part mediated by more feelings of hesitancy towards Project *Wolbachia–*Singapore. This is in line with the health belief model, which posits that a greater perception of threat leads to greater likelihood of performing the protective behavior. It also corroborates with Wong et al.’s [29] empirical finding that dengue prevention practices were less likely to be carried out in neighborhoods with no or low presence of mosquitoes. This observed effect of perceived mosquito density on hesitancy could be because of Singapore’s success in reducing the *Aedes* mosquito population locally [7], such that residents generally face low exposure to mosquitoes and are consequently more sensitive to the presence of mosquitoes. Our finding that those who perceive mosquito bites to be more severe were more hesitant towards Project *Wolbachia*–Singapore is also in line with the study by Kittayapong et al. [13], which showed that those who were afraid of mosquito bites were unwilling to have male *Wolbachia*-carrying mosquitoes released in their homes. Taken together, the data suggest that it would be useful to explore ways of attenuating the effects of the perceived increase in mosquito numbers and perceived severity of mosquito bites so as to strengthen existing communication efforts and reduce hesitancy towards Project *Wolbachia*–Singapore. For example, it may be interesting to compare the effect of using weaker or stronger messaging in preempting residents about the increase in mosquito density with Project *Wolbachia*–Singapore and highlighting ways to prevent mosquito bites, to understand how hesitancy towards the project may be reduced without resulting in complacency against dengue prevention.

Our study has a few limitations. Firstly, as this study design is a cross-sectional survey, we are unable to draw causal inferences. However, future research can draw on the correlations identified in this research to further explore for causal relationships, such as the effect of varying the types and intensity of communications to increase knowledge about *Wolbachia* technology on one’s attitude and intentions to carry out reactive behaviors across time. Such research will more effectively inform how the authorities can cultivate better public attitudes towards Project *Wolbachia–*Singapore, so as to leverage on its demonstrated benefits. Secondly, it should be noted that the questions used to assess social responsibility in this study are limited to Project *Wolbachia*–Singapore. This narrower scope in turn limits the interpretation of findings in this study. Future studies should also use broader measures of social responsibility that are not restricted to a specific project, as that can help to provide insights that are more applicable to other contexts. Thirdly, the data captured in this study only reflects the perspectives and self-reported behaviors of members of the public who agreed to the survey, which may not be representative of the general population at large. Nonetheless, the correlations identified in this study have extended existing knowledge about the less well-examined field of using male *Wolbachia-Aedes* mosquitoes to reduce dengue, and provided the foundations for further studies, especially in the Singapore context. In particular, this research can form the base for future longitudinal studies on how the public perception of Project *Wolbachia–*Singapore changes with increased exposure to the initiative.

## Conclusion

Attitudes and behaviors in relation to Project *Wolbachia–*Singapore are affected by a multitude of factors beyond just general knowledge. While it is encouraging to know that residents in the affected release sites are generally receptive to the project, as the project continues to expand, there is a need to also address the concerns of those who are more hesitant towards the project. This study has revealed various possible ways of reducing hesitancy towards Project *Wolbachia*–Singapore. Future outreach efforts should not only continue to emphasize the role of social responsibility, but also explore ways of tailoring education efforts to those who are less knowledgeable about Project *Wolbachia*–Singapore. In addition, efforts should be made to attenuate the effects of perceived increase in mosquito density and perceived severity of mosquito bites through public communication and engagement platforms.
